# Stepwise Approach for Transvenous Lead Extraction in a Large Single Centre Cohort

**DOI:** 10.3390/jcm12247613

**Published:** 2023-12-11

**Authors:** Axel Kloppe, Julian Fischer, Assem Aweimer, Dominik Schöne, Ibrahim El-Battrawy, Christoph Hanefeld, Andreas Mügge, Fabian Schiedat

**Affiliations:** 1 Department of Cardiology and Angiology at Marienhospital Gelsenkirchen, Academic Hospital of the Ruhr University Bochum, 45886 Gelsenkirchen, Germany; axel.kloppe@ruhr-uni-bochum.de (A.K.); j.fischer@st-augustinus.eu (J.F.); d.schoene@st-augustinus.eu (D.S.); 2 Department of Cardiology and Angiology at University Hospital Bergmannsheil Bochum, Ruhr-University Bochum, 44789 Bochum, Germany; assem.aweimer@ruhr-uni-bochum.de (A.A.); ibrahim.elbattrawy2006@gmail.com (I.E.-B.); andreas.muegge@ruhr-uni-bochum.de (A.M.); 3 Department of Molecular and Experimental Cardiology, Institut für Forschung und Lehre (IFL), Ruhr-University Bochum, 44801 Bochum, Germany; 4 Department of Cardiology at Katholische Kliniken Bochum, Ruhr University Bochum, 44791 Bochum, Germany

**Keywords:** transvenous lead extraction, cardiac electrical devices

## Abstract

Background: Infection, lead dysfunction and system upgrades are all reasons that transvenous lead extraction is being performed more frequently. Many centres focus on a single method for lead extraction, which can lead to either lower success rates or higher rates of major complications. We report our experience with a systematic approach from a less invasive to a more invasive strategy without the use of laser sheaths. Methods: Consecutive extraction procedures performed over a period of seven years in our electrophysiology laboratory were included. We performed a stepwise approach with careful traction, lead locking stylets (LLD), mechanical non-powered dilator sheaths, mechanical powered sheaths and, if needed, femoral snares. Results: In 463 patients (age 69.9 ± 12.3, 31.3% female) a total of 780 leads (244 ICD leads) with a mean lead dwelling time of 5.4 ± 4.9 years were identified for extraction. Success rates for simple traction, LLD, mechanical non-powered sheaths and mechanical powered sheaths were 31.5%, 42.7%, 84.1% and 92.6%, respectively. A snare was used for 40 cases (as the primary approach for 38 as the lead structure was not intact and stepwise approach was not feasible) and was successful for 36 leads (90.0% success rate). Total success rate was 93.1%, clinical success rate was 94.1%. Rate for procedural failure was 1.1%. Success for less invasive steps and overall success for extraction was associated with shorter lead dwelling time (*p* < 0.001). Major procedure associated complications occurred in two patients (0.4%), including one death (0.2%). A total of 36 minor procedure-associated complications occurred in 30 patients (6.5%). Pocket hematoma correlated significantly with uninterrupted dual antiplatelet therapy (*p* = 0.001). Pericardial effusion without need for intervention was associated with long lead dwelling time (*p* = 0.01) and uninterrupted acetylsalicylic acid (*p* < 0.05). Conclusion: A stepwise approach with a progressive invasive strategy is effective and safe for transvenous lead extraction.

## 1. Introduction

As implantation rates of cardiac implantable electronic devices (CIED) have increased over the past decades, the number of complications associated with these devices has increased [1,2]. Possible indications to perform transvenous lead extraction (TLE) are infections, lead dysfunctions and system upgrades [2]. Due to the high burden of electrodes (>5 electrodes via superior vena cava), the number of extractions due to tricuspid regurgitation is also increasing [3,4,5]. With an increasing age of patients presenting for TLE, there are increasing comorbidities, and lead dwelling time is increasing. There are different access methods, and many tools are available to perform lead extraction [6]. According to guidelines, there are no directives to guide operators in their choice of methods and tools for extraction. Therefore, centres and operators have their own preferred strategies [2,7]. The aim of this study is to report the success and associated complications for our stepwise approach, from a less to a more invasive strategy for transvenous lead extraction without the use of a laser sheath in a large single centre cohort.

## 2. Methods

### 2.1. Study Population

Between January 2011 and August 2018, a total of 463 consecutive patients have been referred to our centre for a transvenous lead extraction procedure. We obtained written informed consent. The study protocol conforms to the ethical guidelines of the 1964 Declaration of Helsinki and its later amendments. It was approved by the local ethics committee. All patients received a 12-lead electrocardiogram (ECG), CIED interrogation, thoracic X-ray, transthoracic echocardiography (TTE) and laboratory tests before the procedure. If an infection was present, transoesophageal echocardiography (TOE) was performed as well. Medical history was obtained. Patients with leads implanted for less than 6 months were not included.

### 2.2. Procedure Setting and Sedation

Two experienced operators performed every procedure in our electrophysiology (EP) laboratory. Single or dual antiplatelet therapy has been continued as indicated, oral anticoagulation has been interrupted at least 24 h before the procedure and continued not earlier than 24 h after the procedure. In patients receiving Vitamin-K antagonists (e.g., Phenprocoumon), the procedure was performed if INR (International Normalised Ratio) was lower than 2.0. Procedures were performed under deep sedation (DS), which was classified according to the American Society of Anaesthesiology as breathing spontaneously with unresponsiveness to vocal stimuli and tolerating an oropharyngeal airway [8]. Our deep sedation strategy for TLE will be reported elsewhere.

During the procedure, an anaesthesiologist and cardiac surgeons were available within 2 min as back-up. During the time of the procedure, they had no other duties. Equipment for conversion to GA or open-heart surgery was present in the EP laboratory, including a heart-lung machine. Monitoring was performed with continuous ECG, oxygen saturation and invasive blood measurements. Arterial blood gas analyses were performed once an hour, and more frequently if needed. All patients received a central venous catheter before the procedure and a femoral sheath with a stiff wire was advanced in any jugular, brachiocephalic or subclavian vein to enable rapid deployment of an endovascular occlusion balloon in case of superior vena cava injury. This has been a routinely established procedure since the introduction of the occlusion balloon in 2016. TOE was performed in patients with passive fixation right atrium (RA) or right ventricular (RV) electrodes. In all other cases, TTE with probe was placed in two sterile sheaths, sterile acoustic gel was available, and focused echocardiographic evaluation for pericardial effusion was performed immediately after extraction of every lead. If there was no pericardial effusion, TTE was repeated two hours after the procedure and the next day. If pericardial infusion was present, it was drained or checked regularly depending on the relevance and hemodynamic stability.

Patients received continuous norepinephrine if mean arterial pressure dropped below 60 mmHg. Oxygen was applied via nose canula to maintain a peripheral saturation level >90%.

A temporary right ventricular pacemaker lead was placed in Pacemaker dependent patients via an additional femoral venous access, and if re-implantation needed to be postponed, a transcutaneous screw-in right ventricular lead was inserted through the jugular vein and connected to an external pacemaker after extraction.

### 2.3. Lead Extractions

For lead extraction, a superior approach was initially chosen for every patient. After application of mepivacaine, the device pocket was opened and the leads, including their sleeves, were fully exposed. The leads were dissected from all tissue, the sleeves were removed, and, if possible, an active fixation mechanism was retracted.

If the lead structure was intact according to X-ray, a standard stylet was inserted, and simple traction was performed first. If this was not successful, the lead structure was exposed by cutting the lead distal to the connector pin. A lead locking device (LLD EZ, Spectranetics, Colorado Springs, CO, USA) was inserted and traction was repeated. If this was unsuccessful, a telescopic mechanical non-powered polypropylene sheath system (Byrd Dilator Sheaths, Cook Medical, Bloomington, IN, USA; Sight rail, Spectranetics, Colorado Springs, CO, USA) was used. If extraction was not possible due to severe adhesions, the dilator sheaths were removed and a mechanical rotating sheath (Tight rail, Spectranetics, Colorado Springs, CO, USA; Evolution RL, Cook Medical, Bloomington, IN, USA) was advanced. If lead structure was not intact, an LLD could not be advanced into the lead, a significant lead fragment remained, or if it still was not successful and lead extraction was necessary due to infection, a 16 French femoral access was used to deploy a double loop snare (Needle Eye Snare, Cook Medical, Bloomington, IN, USA). If extraction of a lead was not mandatory as the main goal for extraction has been reached (e.g., system upgrade possible), it was possible for the operators to end the procedure after each step to avoid unnecessary risks.

### 2.4. Definitions

Success of lead extraction was recorded according to the Heart Rhythm Society (HRS) and European Heart Rhythm Association (EHRA) guidelines for TLE. Complete success was defined as removal of all parts of all targeted leads. Clinical success was defined as retention of a small part < 4 cm of at least one targeted lead without risk of embolic events, perpetuation of infection or increased risk of perforation. Major and minor complications were defined according to the HRS and EHRA guidelines, which are identical in this regard [4,5]. Chronic renal insufficiency was defined as at least a moderate reduction in glomerular filtration rate (GFR) < 60 mL/min/1.73 m^2^ over a period > 3 months.

### 2.5. Data Collection

Data has been collected continuously and checked afterwards with clinical information system.

### 2.6. Statistical Analysis

All statistical analysis was performed using IBM SPSS Statistics version 24.0.0 on Mac.

Categorial variables were expressed as frequencies and percentages (normal distribution) or median and interquartile range (non-normal distribution) and compared by chi-square test or Fisher’s exact test. Continuous variables were stated as mean ± standard deviation and compared with unpaired *t*-test/ANOVA for normally distributed variables and Mann–Whitney *U*-test for non-normally distributed variables.

Correlations have been evaluated by Pearson’s or Spearman’s Rho. All statistical analyses were two-sided, and *p* < 0.05 was considered statistically significant.

## 3. Results

### 3.1. Baseline Characteristics

A total of 463 patients were included in this study over a period of seven years. Mean age was 69.9 ± 12.3 years, and 31.3% (*n* = 145) were female. Underlying heart disease was ischemic cardiomyopathy in 119 patients (25.7%), and non-ischemic cardiomyopathy in 144 patients (31.1%). A total of 97 patients (21.0%) had undergone previous open-heart surgery. Mean left ejection fraction was 45.3 ± 12.6%. The study population consisted of 205 pacemaker systems (44.4%), 254 implantable cardioverter defibrillator (ICD) systems (54.8%) and four (0.8%) cardiac contractility modulation (CCM) systems. In total, 126 (29.8%) were CRT systems. Mean lead dwelling time was 5.4 ± 4.9 years. Indication for extraction were infection (33.7%), lead malfunction or dislocation (52.7%), lead perforation (9.7%) and system upgrade (3.9%), respectively. Continuous acetylsalicylic acid (ASA) intake was present in 196 patients (42.3%), and continuous dual antiplatelet therapy (DAPT) in 37 patients (8.0%). Baseline patient characteristics are listed in Table 1.

### 3.2. Procedure and Success Rate

Of 1025 leads in 463 patients, 780 leads were identified for extraction. Of these, *n* = 447 (57.3%) were pacemaker leads, *n* = 244 (31.3%) defibrillator leads and *n* = 89 (11.4%) coronary sinus (CS) leads. A total of 126 leads (16.2%) were passive fixation RA and RV leads. Procedure time was 103.4 ± 68.8 min. In 87 patients (18.8%), more than two leads were identified for extraction.

Extraction with standard stylet and simple traction was successful in 246 leads (31.5% success rate). For 38 leads, the stepwise approach was not further pursued as lead components were not intact or an LLD could not be inserted into the lead. In these cases, the procedure was continued via a femoral access to extract the lead with a snare. Extraction with LLD and simple traction was used in 496 leads and successful in 212 leads (42.7% success rate). Procedure ended unsuccessfully after this step at the operator’s discretion to avoid higher procedural risks for 39 leads in which extraction was initially planned.

Mechanical non-powered sheaths were used for 246 leads and were successful in 207 leads (84.1% success rate). Extraction ended after this step at the operator’s discretion in 11 leads in whom total extraction was not successful. A mechanical rotation sheath was used for 27 leads and successful for 25 leads (92.6%). There was no significant difference in performance between the different manufacturers used for mechanical non-powered and mechanical rotation sheaths used.

**Figure 1:**
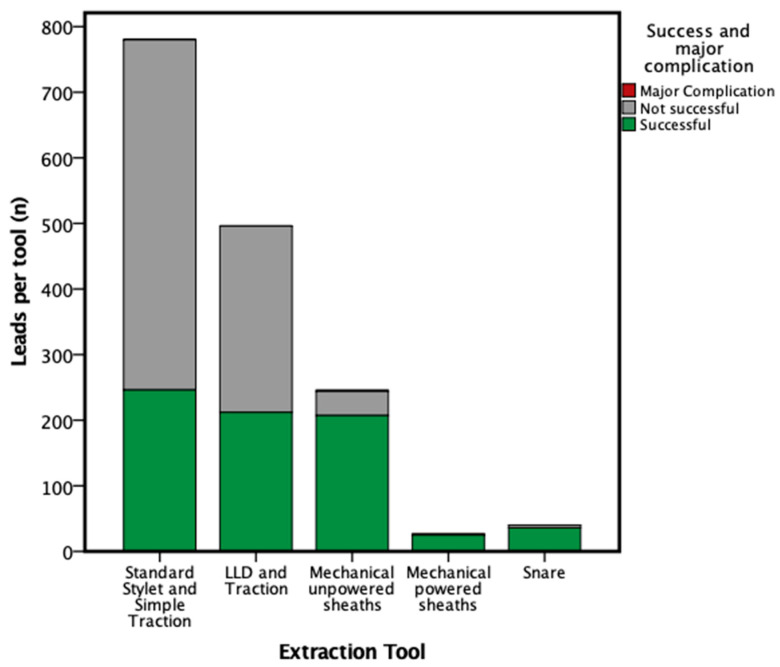
Stacked bar chart illustrating success per tool and major complication associated with the tool.

For a total of 40 cases, a snare has been used. Snare was successful in 36 leads (90.0%). A remaining lead fragment < 4 cm was present in eight patients (1.7%). Success rate for each tool is illustrated in Figure 1.

Total success rate was 93.1% (*n* = 726/780), and clinical success rate was 94.1% (*n* = 734/780). For 50 leads (6.4%) the extraction procedure was terminated early without total extraction success at the operator’s discretion as the need for extraction did not outweigh the risks of continued extraction efforts. Procedural failure was accounted for in five patients (1.1%). Procedural characteristics are summarized in Table 2 and Table 3.

### 3.3. Determinants of Success

**Figure 2:**
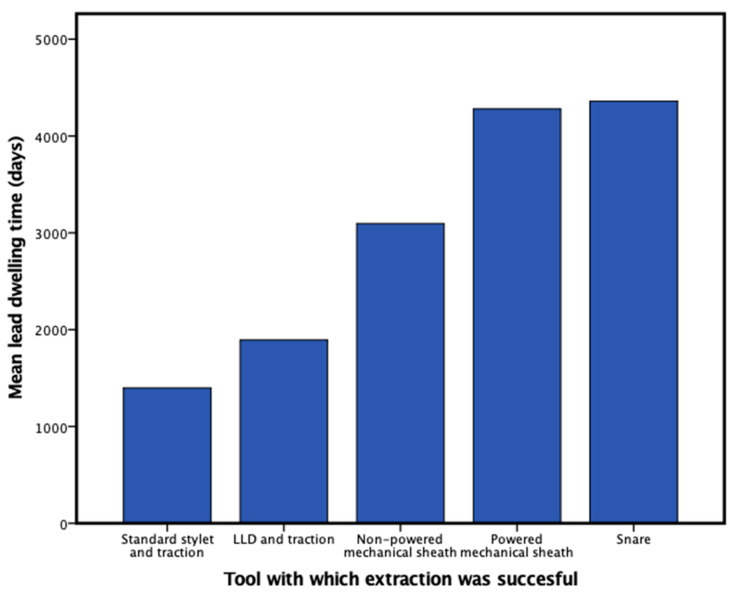
Bar graph illustrating which tool was used for successful extraction in a stepwise approach and mean lead dwelling time.

There was a significant correlation between procedure time and mean lead dwelling time (R = 0.39, *p* < 0.001). Active fixation RA and RV leads were associated with higher extraction success rates for simple traction and for extraction with LLD and traction (*p* < 0.05). There were significant differences in lead dwelling time and success of extraction for the less invasive tools. For standard stylet and simple traction, mean lead dwelling time for successful extraction was 1398.2 ± 725.4 days vs. 3033.4 ± 1398.1 days for unsuccessful extraction (*p* < 0.001). For LLD and traction, mean lead dwelling time for successful extraction was 1894.9 ± 798.7 days vs. 3488.8 ± 1050.4 days (*p* < 0.001) for unsuccessful extraction. For mechanical non-powered sheath, extraction was successful in electrodes with a mean lead dwelling time of 3097.3 ± 1022.4 days vs. unsuccessful in leads with a mean dwelling time of 4269.9 ± 994.5 days (*p* < 0.001). There was no significant difference for success rates at each step between pacemaker, ICD and CS leads and between single- and dual-coil ICD leads. Mean lead dwelling time for successful extraction for each step is illustrated in Figure 2.

## 4. Complications

**Figure 3:**
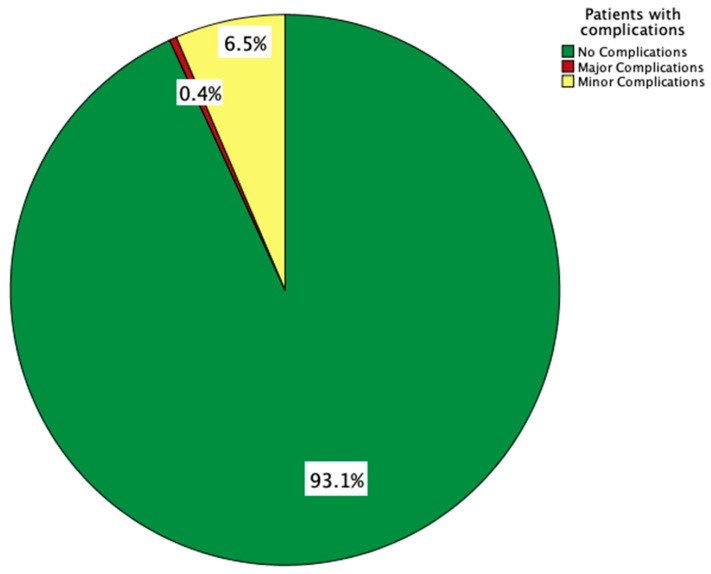
Pie chart illustrating complication rate associated with a stepwise minimal-invasive approach for transvenous lead extraction.

Major complications occurred in two patients (0.4%). Minor complications occurred in 30 patients (6.5%), with a total of 36 minor complications. Some patients experienced more than one minor complication. Complication rates are illustrated in Figure 3 and listed in Table 4.

One patient (0.2%) died during the procedure because of a right ventricular rupture with pericardial tamponade. This was a patient with a single chamber, right ventricular, active fixation, dual-coil ICD lead with a dwelling time of 9.3 years, presenting for extraction because of infection. The complication occurred during extraction with a mechanical non-powered sheath. Even though the procedure was converted into an open-heart surgery within 90 s, the outcome was fatal. The other major complication was a dissection of the superior vena cava that was successfully handled by an occlusion balloon and conversion to open heart surgery in one patient (0.2%). This was a patient with a CRT-D system in whom the dissection occurred during extraction of the LV lead while using a mechanical non-powered sheath. Lead dwelling time of the LV lead was 8.6 years, and overall time from first implant until extraction was 20.6 years.

Most common minor complications were pocket hematoma requiring intervention (*n* = 8, 1.7%) and pericardial effusion without intervention (*n* = 12, 2.6%). Ventricular tachycardia was documented in 18 patients (3.0%), of which 14 occurred at a mean of 2.2 ± 1.8 days after the procedure and none of which were fatal. Some patients experienced more than one complication.

### Determinants of Complications

Renal insufficiency was significantly associated with procedure-related complications (R = 0.109, *p* < 0.05), and history of ventricular tachycardia prior to extraction was significantly associated with an episode of ventricular tachycardia during the first 5 days after the extraction procedure (R = 0.108, *p* < 0.05). Pocket hematoma correlated significantly with uninterrupted DAPT (R = 0.148, *p* =0.001). The presence of pericardial effusion without need for intervention was associated with a long lead dwelling time (R = 0.0121, *p* = 0.01) and uninterrupted ASA (R = 0.103, *p* < 0.05).

## 5. Discussion

Although guidelines for TLE exist, there are no recommendations for a specific or systematic approach [4,5]. Every centre has an individual approach and uses different tools. A systematic approach from a less to a more invasive approach is more time consuming than a primary approach with a powered sheath, but could be associated with lower complication rates. We therefore sought to investigate an approach which systematically starts with a less invasive approach and increases severity step-by-step with a femoral snare as a bail-out strategy. Laser sheaths were not used; even though they have shown the highest success rates for TLE according to a large review by Buiten et al., they are also associated with the highest rates of mortality, as well as major and minor procedure-related complications. Complications due to the use of laser sheaths are even more frequent than with femoral access snares [7]. The use of laser sheaths can often only be cost-effective if used for every procedure in a centre [9,10]. We therefore sought to exclude laser sheaths in this stepwise approach as it did not seem feasible for a real-world stepwise approach.

Simple traction, mostly with a standard stylet inserted, is often used in leads implanted for a short time. The success rate for this less invasive approach in our cohort is comparable to data from the ELECTRa registry (31.5% in our cohort vs. 27.3% in the ELECTRa registry) [2]. Successful extraction with simple traction was associated with shorter lead dwelling time and active fixation leads, as also reported in a review by Buiten et al. [7]. Compared to other studies that reported success rates of 70–93% with simple traction for CS lead, our success rate for CS lead extraction with simple traction was significantly lower, with 25.8% [11,12,13]. Compared to those studies, the lead dwelling time in our cohort was longer (5.4 ± 4.8 years vs. 1.5–3.0 years), and our cohort consisted of a higher number of CRT-D systems compared to CRT-P systems. Longer lead dwelling time and more ICD leads present could lead to more frequent and more severe venous adhesions, meaning lower success rates for CS lead extraction with simple traction. For LLD and traction only, little data on success rates has been reported [7]. Our cohort is one of the largest so far, reporting a detailed analysis for this step. Our success rate is very comparable to studies with similar lead dwelling time and regular use of LLD and traction during the extraction procedure (42.7% in our cohort vs. 37% in data reported by Geselle et al. and Williams et al.) [11,14]. After the first two less invasive steps, we successfully extracted 58.7% of all leads identified for extraction without major complications. As these steps impose little risk with a relatively high success rate, it seems reasonable to advise trying these steps first in the majority of extraction procedures, especially for active fixation leads. With mechanical non-powered sheaths, we successfully extracted 84.1% of the leads that were not extracted during the first two steps. The success rate was lower compared to a very large cohort reported by Kutarski et al., which had a 95% success rate in 2049 patients [15]. Our success rate could be lower as we were able to escalate to the next step if extraction with mechanical non-powered sheaths was difficult. However, the major complication rate was higher in the data reported by Kutarski et al. (1.8% vs. 0.4% in our cohort). The higher complication rate could be due to longer lead dwelling time (86.3 months reported by Kutarski et al. vs. 64.3 months in our study) and a high dedication to successfully extracting leads by only using non-powered mechanical sheaths [15]. Both major complications in our cohort occurred during extraction using non-powered mechanical telescopic sheaths. As non-powered sheaths require manual rotation of the sheaths, an uneven, non-continuous rotation and traction can increase the risk of a major complication. With powered mechanical sheaths, we extracted 92.6% of leads that were not extractable before during the first three steps. Our success rate was higher than data reported by Zsigmond et al. [16]. Lead dwelling time was even longer in our cohort for patients in which a mechanical powered sheath was used (11.7 years in our cohort vs. 9.4 years reported by Zsigmond et al. [16]). Zsigmond et al. compared mechanical powered sheaths with laser powered sheaths as the first line tool and reported a crossover rate due to extraction failure of 19.5% for laser sheaths and 13% for mechanical powered sheaths. A significant number of crossovers were necessary due to severe adhesions at extracardiac levels [16]. Our high success rate could be explained by the stepwise approach having helped detach fibrous adhesions. For different manufacturers, operators did not report any relevant technical difference in handling or usability of the devices. Even though there was little data available to compare different manufacturers, we did not see a trend that would suggest one manufacturer’s sheath system was superior to another. Our stepwise approach appears to be as efficient as a crossover between two very invasive approaches with a lower complication rate (2.2–5.2% major complications and 8.7–12.5% minor complications vs. 0.4% major complications and 6.5% minor complications in our cohort) [16]. Procedural failure was accounted for in 1.1% of our patients. Additional use of laser sheaths could have helped in these cases. In our centre, we referred those patients to our cardiothoracic surgeons. We therefore recommend considering the use of laser sheaths in future stepwise approach extraction procedures.

Our total procedural success rate was lower compared to other large studies [17]. The possibility of ending the extraction procedure at the operator’s discretion if they determined that the major goal of the procedure had been achieved to avoid unnecessary risks, even if leads were abandoned, is a likely explanation. As expected, procedure time was longer in our cohort compared to the ELECTRa registry (83.0 vs. 103.4 min) [2]. However, in our opinion, the lower complication rate outweighs the longer procedure time.

Procedure-related major complication rates were low in our cohort compared to the ELECTRa registry (1.7% vs. 0.4% in our cohort) [2]. The stepwise approach, the possibility to end the extraction procedure at the operator’s discretion if the main extraction goal was achieved, and our experience as a high volume centre could be explanations. This agrees with a study by Bontempi et al. who reported a major complication rate of 0.6% in 973 patients with a stepwise approach [17]. Minor complication rates in our study were comparable to the ELECTRa registry (5.0% vs. 6.5% of patients in our cohort) [2]. Minor complications were associated with comorbidities or medication and not with the extraction strategy in our cohort. In order to avoid pocket hematoma, a short interruption of one antiplatelet agent in DAPT should be considered if possible. An interruption of ASA does not seem necessary as there only was a correlation for pericardial effusion, which did not require an intervention. According to our data, patients with renal insufficiency and history of ventricular arrhythmia need to be monitored closely during and after the procedure. A prolonged ECG monitoring for patients with history of ventricular arrhythmia seems reasonable.

## 6. Limitation

Our single-centre experience does not compare different approaches for lead extraction, and we did not randomize our patients into different approaches. In addition, laser sheaths are often used in many centres and are not included in our stepwise approach. It was possible to terminate the procedure at the operator’s discretion if risks outweighed benefits, which could make it difficult to compare our data presented to other data reported. To further investigate venous obstruction as a possible complicating factor for implantation of new leads or even TLE, we changed our protocol; currently, we perform venography before all TLE procedures. If there is a plan to reimplant leads after TLE, venography can be particularly helpful in identifying patients with severe or total lead related venous obstruction. Severe or total venous occlusion can be found in 42.4% of the 3002 patients referred for TLE, according to Czajkowski et al. [18]. This helps to plan the extraction strategy, enabling new lead implants at the same site and preserving the vasculature of the opposite site for other procedures in the future.

## 7. Conclusions

A stepwise approach with a progressive invasive strategy is effective and safe for transvenous lead extraction. Less invasive steps are not only very effective for extracting leads with a short dwelling time, these steps can also help in a stepwise approach to successfully explant leads with a long dwelling time as each approach seems to detach fibrous adhesions. A stepwise approach can reduce the rate of major complications, even though procedure time might be prolonged.

## Figures and Tables

**Table 1:** Baseline characteristics and acute measurement results (*n* = 463). BMI = Body Mass Index, COPD = chronic obstructive pulmonary disease, NYHA = New York Heart Association class, LV-EF = left ventricular ejection fraction, DAPT = dual antiplatelet therapy, DOAK = direct oral anticoagulant, LMWH = Low molecular weight heparin, ICD = implantable cardiac defibrillator, CCM = cardiac contractility modulation system.

Characteristics	Value
Age (years)	69.9 ± 12.3
Sex, female *n* (%)	145 (31.3)
BMI (kg/m^2^)	27.5 ± 5.6
Weight (kg)	82.4 ± 19.5
Renal Insufficiency, *n* (%)	168 (36.3)
Stage I, *n* (%)	8 (1.7)
Stage II, *n* (%)	36 (7.8)
Stage III, *n* (%)	97 (21.0)
Stage IV, *n* (%)	14 (3.0)
Stage V with dialysis, *n* (%)	14 (3.0)
Arterial hypertension, *n* (%)	416 (89.9)
Diabetes mellitus, *n* (%)	145 (31.1)
COPD, *n* (%)	95 (20.5)
Coronary artery disease, *n* (%)	253 (54.6)
Heart failure, *n* (%)	254 (54.9)
Ischemic, *n* (% total; % Heart failure)	119 (25.7; 45.2)
NYHA III/IV, *n* (%)	154 (33.4)
Atrial fibrillation, *n* (%)	218 (47.1)
History of ventricular arrhythmia, *n* (%)	150 (32.4)
History of open-heart surgery, *n* (%)	97 (21.0)
LV-EF (%)	45.3 ± 12.6
**Medication**	
Aspirin, *n* (%)	196 (42.3)
DAPT, *n* (%)	37 (8.0)
Phenprocoumon, *n* (%)	154 (33.3)
DOAK, *n* (%)	53 (11.4)
LMWH, *n* (%)	23 (5.0)
**System**	
Pacemaker, *n* (%)	205 (44.4)
Single Chamber, *n* (%)	40 (8.6)
Dual Chamber *n* (%)	150 (32.4)
CRT-P, *n* (%)	15 (3.2)
ICD, *n* (%)	254 (54.8)
Single Chamber, *n* (%)	84 (18.1)
Dual Chamber, *n* (%)	47 (10.2)
CRT-D, *n* (%)	123 (26.6)
Primary prophylactic, *n* (%)	189 (74.4)
Secondary prophylactic, *n* (%)	65 (25.6)
CCM, *n* (%)	4 (0.9)
**Location of device**	
Left sided system, *n* (%)	395 (85.3)
Subcutan, *n* (%)	147 (31.7)
Subfascial, *n* (%)	158 (34.1)
Submuscular, *n* (%)	158 (34.1)
**Leads**	
Total number of implanted leads (*n*)	1025
Implanted Leads per patient	
1, *n* (%)	107 (23.1)
2, *n* (%)	186 (40.2)
3, *n* (%)	142 (30.7)
4, *n* (%)	22 (4.8)
5, *n* (%)	5 (0.9)
6, *n* (%)	6 (0.4)
Mean Lead dwelling time, months	64.3 ± 57.8
**Reason for extraction**	
Infection, *n* (%)	156 (33.7)
Pocket associated, *n* (%)	100 (21.6)
Endoplastitis, *n* (%)	45 (9.7)
Endocarditis, *n* (%)	11 (2.4)
Lead malfunction or dislocation, *n* (%)	244 (52.7)
Lead perforation, *n* (%)	45 (9.7)
System upgrade, *n* (%)	18 (3.9)

**Table 2:** Procedural characteristics. CS = coronary sinus.

Characteristics	Value
Procedure time (min)	103.4 ± 68.8
Fluoroscopy time (min)	9.3 ± 13.1
Leads for extraction, *n* (% of total lead burden)	780 (76.1)
Leads per patient for extraction	
1, *n* (%)	252 (54.4)
2, *n* (%)	124 (26.8)
3, *n* (%)	70 (15.1)
4, *n* (%)	16 (3.5)
5, *n* (%)	0
6, *n* (%)	1 (0.2)
Pacemaker leads, *n* (%)	447 (57.3)
Pace/Sense leads, *n*	32
ICD leads, *n* (%)	244 (31.3)
Single coil, *n*	201
Dual coil, *n*	43
CS, *n* (%)	89 (11.4)
Fixation mode	
Screw (active), *n* (%)	565 (72.4)
Passive, *n* (%)	126 (16.2)
CS passive, *n* (%)	89 (11.4)
Leads for extraction per chamber	
Total Atrial, *n* (passive fixation *n*, %)	228 (25, 11.0)
1, *n*	221
2, *n*	7
Atrial mean lead dwelling time, months	60.4 ± 54.7
Total RV, *n* (passive fixation *n*, %)	463 (101, 21.8)
1, *n*	415
2, *n*	44
3, *n*	4
Ventricular mean lead dwelling time, months	54.7 ± 52.7
Total CS, *n* (passive fixation *n*, %)	89 (89, 100)
1, *n*	88
2, *n*	1
CS mean lead dwelling time, months	47.1 ± 36.8
Total success rate, *n* (%)	726 (93.1)
Clinical success rate, *n* (%)	734 (94.1)
Screw in Electrode until Re-implant, *n* (%)	32 (6.9)
Transesophageal Echocardiography during procedure, *n* (%)	76 (16.4)
Length of hospital stay (days)	6.3 ± 8.2

**Table 3:** ✓ = successful, X = not successful, *n* = number of times device used to achieve extraction by this method.

	Atrial, *n* (% Success)		RV, *n* (% Success)		LV, *n* (% Success)		Total, *n* (% Success)		
	✓	*n*	✓	*n*	✓	*n*	✓	X	*n*
Standard stylet + light traction	81	228 (35.5)	142	463 (30.7)	23	89 (25.8)	246	534	780 (31.5)
Lead locking device (LLD) + light traction	58	137 (42.3)	130	298 (43.6)	24	61 (39.3)	212	284	496 (42.7)
Mechanical non-powered sheath	65	74 (87.8)	118	142 (83.1)	24	30 (80.0)	207	39	246 (84.1)
Byrd dilator sheath		47		93		26			166
Sight rail sheath		27		49		4			80
Mechanical powered sheath	7	7	16	17	2	3	25	2	27 (92.6)
Evolution RL	3	3	8	8	1	2	12	1	13
Tight rail	4	4	8	9	1	1	13	1	14
Snare	9	10	22	25	5	5	36	4	40 (90.0)
Remaining electrode									
0 cm		220		428		78			726
<4 cm		3		4		1			8
>4 cm		5		31		10			46
Clinical success		223/228		432/463		79/89	94.1%		734/780
Total success		220/228		428/463		78/89	93.1%		726/780

**Table 4:** Complications associated with the procedure.

Complications	Intra—OP	Post—OP	Total
**Major—Complications according to guidelines**	2 (0.4)		**2 (0.4)**
**Death, *n* (%)**	1 (0.2)		1 (0.2)
**Reason: Ventricular rupture, *n* (%)**	1 (0.2)		
**Vascular laceration, *n* (%)**	1 (0.2) _1_		1 (0.2)
**+Endovascular occlusion balloon, *n* (%)**	1 (0.2)		
**Respiratory arrest, *n***			0
**Cerebrovascular accident, *n***			0
**Pericardial Tamponade requiring intervention, *n* (%)**			0
**Hemothorax requiring intervention, *n***			0
**Cardiac arrest, *n***			0
**Thromboembolism requiring intervention**			0
**Flail tricuspid valve leaflet requiring intervention, *n***			0
**Massive pulmonary embolism, *n***			0
**Minor—Complications according to guidelines**			
**Overall minor complications**			**36**
**Patients with minor complications**			**30 (6.4)**
**Pericardial effusion without intervention, *n* (%)**		12 (2.6)	12 (2.6)
**Detected (hours after extraction)**		19.2 ± 33.6	
**Associated with long-dwelling time**		R = 0.121, *p* = 0.01	
**Pocket hematoma requiring intervention, *n* (%)**		8 (1.7)	8 (1.7)
**Association to DAPT**		R = 0.148, *p* = 0.001	
**Transfusion required, *n* (%)**		4 (0.9)	4 (0.9)
**Venous thrombosis requiring medical intervention, *n* (%)**		1 (0.2)	1 (0.2)
**Vascular repair at entry site, *n***			0
**Migrated lead fragment without sequelae, *n* (%)**	1 (0.2)		1 (0.2)
**Bleeding requiring blood transfusion, *n* (%)**		10 (2.2)	10 (2.2)
**AV fistula requiring intervention, *n***			0
**Pneumothoraxes requiring chest tube, *n* (%)**		2 (0.4)	
**Detected (hours after extraction)**		28.8 ± 52.8	
**Worsening tricuspid valve function, *n***			0
**Pulmonary embolism**			0
**Other complications, not defined according to guidelines**			
**Sedation associated, *n* (%)**			5 (1.1)
**Hypotension, *n***	2		
**Respiratory Insufficiency, *n***	1	2	
**Nosocomial pneumonia, *n* (%)**		4 (0.9)	4 (0.9)
**Ventricular tachycardia, *n* (%)**	4 (0.9)	14 (3.0)	18 (3.9)
**Days after extraction**		2.2 ± 1.8 _2_	

_1_ Brachiocephalic vein with hemopericardia, _2_ Days after Extraction: First postoperative day: 6, 2 days: 3, 3 days: 4, 5 days: 1.

## Data Availability

All data can be made available by Fabian Schiedat upon request.

## Abbreviations

ASA	acetylsalicylic acid
BMI	body mass index
CCM	cardiac contractility modulation
CIED	cardiac electrical devices implanted
COPD	chronic obstructive pulmonary disease
CPAP	continuous positive airway pressure
CRT-D	cardiac resynchronization therapy defibrillator
CRT-P	cardiac resynchronization therapy pacemaker
CS	coronary sinus
DS	deep sedation
ECG	electrocardiogram
EHRA	European heart rhythm society
EP	electrophysiology
GA	general anaesthesia
GFR	glomerular filtration rate
HRS	heart rhythm society
ICD	implantable cardioverter defibrillator
LLD	lead locking device
LV-EF	left ventricular ejection fraction
Min	minutes
NYHA	New York Heart Association
RA	right atrium
RV	right ventricular
TAVR	transcatheter aortic valve replacement
TLE	transvenous lead extraction
TOE	transoesophageal echocardiography
TTE	transthoracic echocardiography
